# Potential generation of nano-sized mist by passing a solution through dielectric barrier discharge

**DOI:** 10.1038/s41598-022-14670-4

**Published:** 2022-06-22

**Authors:** Ryosuke Watanabe, Shiori Tanaka, Godai Miyaji, Daisuke Yoshino

**Affiliations:** 1 Faculty of Engineering, Tokyo University of Agriculture and Technology, 2-24-16 Naka-cho, Koganei, Tokyo 184-8588 Japan; 2 Graduate School of Engineering, Tokyo University of Agriculture and Technology, 2-24-16 Naka-cho, Koganei, Tokyo 184-8588 Japan; 3 Graduate School of Bio-Applications and Systems Engineering, Tokyo University of Agriculture and Technology, 2-24-16 Naka-cho, Koganei, Tokyo 184-8588 Japan; 4 Institute of Engineering, Tokyo University of Agriculture and Technology, 2-24-16 Naka-cho, Koganei, Tokyo 184-8588 Japan

**Keywords:** Mechanical engineering, Biomedical engineering, Electrical and electronic engineering

## Abstract

Plasma medicine, a therapeutic technology that uses atmospheric-pressure plasma, is attracting much attention as an innovative tool for the medical field. Most of the plasma biomedical tools use direct effects, such as heat, optical stimulation, and reactive chemical species, on the lesion. Nanoparticulation techniques using indirect action by plasma, i.e., generation of electric fields, have the potential to be applied to promote transdermal absorption, where drugs pass through the barrier function of skin and penetrate into internal tissues. Here, we show a method to directly generate the nano-sized mist by passing a solution through the dielectric barrier discharge. This method enables us to produce the mist potentially in the nanometer size range for both water-based and oil-based solutions. Ease of mist generation was influenced by the plasma-induced changes in physical and chemical characteristics, including electrical conductivity, viscosity, and chemical species. We anticipate the developed method for nano-sized mist generation to provide a technique in the applications of the transdermal absorption system, including those related to pharmaceuticals and cosmetics.

## Introduction

Plasma in atmospheric pressure is a key technology supporting many innovations in industrial applications because of its generation of electric fields, optical radiation, heat, and reactive chemical species^1–3^. Recently, the plasma systems have been explored for application in the fields of medicine and biology as an effective tool for wound healing^4,5^ or cancer treatment^6,7^. Those biomedical applications are mainly based on the direct action of plasma-derived physical and chemical stimuli (i.e., heat, optical stimulation, and reactive oxygen and nitrogen species) on the target. The plasma application to a lesion in the body is no less surgically demanding for the patient, although it is better when the lesion is exposed, as in the case of burns, skin diseases, and wounds.

Recently, new developments in transdermal absorption systems have been anticipated as a medical technology that reduces systemic load. Transdermal absorption can be enhanced by directly irradiating plasma onto living tissue^8^. On the other hand, if drug penetration can be accelerated by allowing plasma to act on the drug itself, the scope of this application will expand to the respiratory and digestive organs, in addition to the skin, which is easily irradiated directly by plasma. In order to enhance the transdermal penetration of drugs, their nanoparticulation is important^9,10^. Nanotechnology delivery systems make it possible for drugs to pass through the skin barrier and reach the target area in a stable manner with long-lasting efficacy^11^. Plasma-generated nanoparticles^12,13^ may be used in these delivery systems. Whereas they also have the disadvantage of requiring complex chemical processes. Further progress in transdermal drug delivery could be made if nanoparticles could be produced directly from aqueous or oil-based solutions in which the drug is dissolved by a simple process. Electrostatic spray is known as a technique for nanoparticulation of the solutions (i.e., generation of nano-sized mist) using electrical effects^14,15^. Micro- or nano-sized mists produced by electrostatic spray have been applied in a wide range of fields, including pesticide spraying, water purification, and seawater desalination^16^. This technology is expected to facilitate transdermal absorption by miniaturizing the drug solution. Electrostatic spray requires a closed circuit with the target side grounded and a capillary to which a high voltage is applied, making it unsafe for biological applications. On the other hand, a dielectric barrier discharge with the closed circuit can be used to impart sterilization and other effects derived from reactive chemical species generated by the plasma^17^. However, it is difficult to generate liquid particles only by the dielectric barrier discharge, which requires another mist generation mechanism such as an ultrasonic transducer.

**Figure 1:**
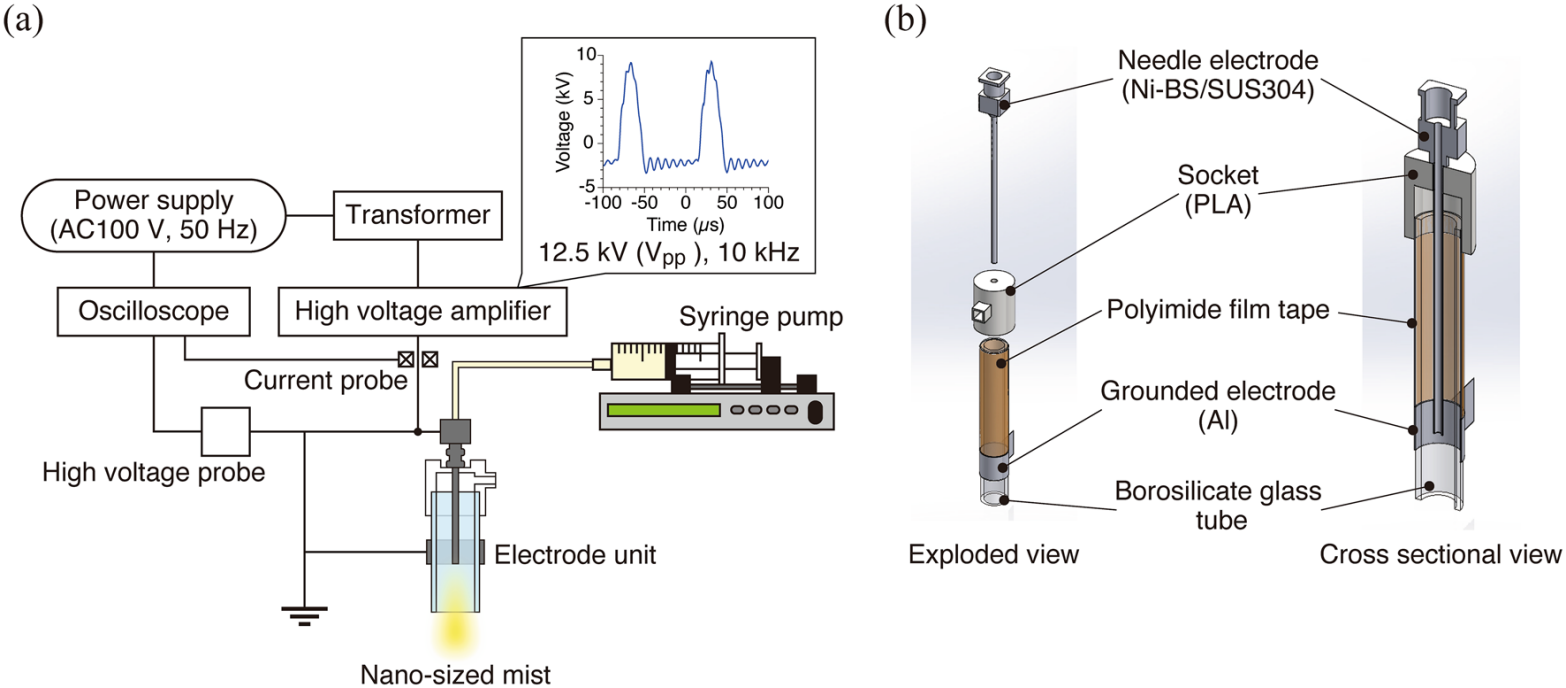
Nano-sized mist generator using DBD. (**a**) Configuration of the generator. (**b**) Electrode design.

In this study, we developed a method to directly generate nano-sized mist by passing a solution through the dielectric barrier discharge (DBD; Fig. 1). We verified mist generation for three types of solutions, including the water-based and oil-based solutions, focusing on the characteristics of the plasma discharge. We then investigated the changes in the physical and chemical characteristics of each solution by plasma treatment. The findings suggest that the electrical conductivity of the solution influenced the efficiency of the mist generation. Our developed method can atomize various types of solutions without grounding the target being sprayed.

## Methods

### Electrode design and plasma generation

Nano-sized mist generator is composed of a high voltage amplifier (Logy Electric, LHV-10AC-TL), a transformer (AS ONE, RSA-10), an airflow pump (AS ONE, EAP-01), a syringe pump (KD Scientific, Legato 110), and a borosilicate glass tube electrode unit (Fig. 1a). Atmospheric pressure plasma of the DBD mode was, in principle, generated in the glass tube with outer and inner diameters of 8 mm and 6 mm, respectively (Fig. 1b). The pulsed voltage was applied to a syringe needle (Tsubasa Industry Co., 16G × 50) placed in the center of the glass tube. An aluminum electrode attached on the outside across the tube was grounded. The outer surface of the glass tube was covered with polyimide film tape to prevent the plasma discharge on the outside of the electrode unit. The applied voltage was 12.5 kV from peak (-3.2 kV) to peak (+ 9.3 kV), with a frequency of 10 kHz, which was determined using a high-voltage probe (Tektronix, P6015A) and an oscilloscope (Teledyne Lecroy, WaveSurfer 3024Z). The gas atmosphere can be controlled by introducing a specific gas through an inlet on the side of the socket. However, in this study, we conducted the experiment under atmospheric pressure environment without gas inflow.

### Observation of plasma emission and its optical characteristics

Light emitted from the plasma in the electrode during voltage application was captured using a digital camera (Fujifilm, X-T4) equipped with a macro lens (Fujifilm, Fujinon XF80mm F2.8 R LM OIS WR Macro). The shutter speed, ISO sensitivity, and the diaphragm of the camera were set to 1/60 s, 2500, and 2.8, respectively. Emission spectra were analyzed using a high-resolution spectrometer (Ocean Insight, HR4000CG-UV-NIR). The range of analyzed wavelengths was from 200 to 1100 nm. The distance from the tip of the electrode to the spectrometer was 50 mm. Each integration time was set to 10 s, and the spectra were calculated from the accumulation of two measurements.

### Generation and visualization of nano-sized mist

Ultrapure water (UPW), phosphate buffered saline (PBS; Nissui, 05913), and castor oil (Fujifilm Wako Pure Chemical Corporation, 034-01586) were used to generate nano-sized mist for a wide range of applications in medicine and cosmetics. The solution was infused into the needle electrode using the syringe pump (Fig. 1a). The in-flowed solution was charged as it passed through the needle electrode, and interacted with the plasma at the tip of the needle to form a nano-sized mist. The generated nano-sized mist was transported outside the electrode unit by airflow from the pump. The infusion rate of the solution was 10, 50, 100, 500, or 1000 µL/min.

Dynamics in the nano-sized mist generated by plasma were visualized by using the digital camera with the macro lens or a microscope lens [Mitutoyo, M Plan Apo 10 × (NA = 0.28) or M Plan Apo 100 × (NA = 0.7)] and a line laser module (Civil laser, 520 nm, 50 mW). The laser module was placed at a distance of 60 mm lateral to the electrode, and the laser sheet with thickness of about 1 mm was incident on a vertical section through the center of the electrode. The focus of the camera was set at 8.0 mm for the macro lens and 2.5 mm for the microscope lens from the glass tube. Movies were captured at the frame rate of 240 fps, ISO sensitivity of 2500, and the diaphragm of the camera of 2.8 (macro lens). High-speed imaging was performed with the shutter speed of 1/8000 s, ISO sensitivity of 8000 and the diaphragm of the camera of 0.0 (microscope lens) for visualization of mist particles.

### Chemical characterization of nano-sized mist

Dissolved chemicals in each solution were detected on the basis of absorbance measurements. We focused on the production of hydrogen peroxide, nitrite ion, and nitrate ion because these species are known to be generated by plasma-liquid interactions^18^. Concentrations of hydrogen peroxide, nitrite ions, and nitrate ions in the solutions (UPW and PBS) were measured based on potassium iodide colorimetric assay (Kyoritsu Chemical-Check Lab, WAK-H2O2(C)), naphthyl ethylenediamine colorimetric assay (Kyoritsu Chemical-Check Lab, WAK-NO2, WAK-NO3), and Griess assay (Kyoritsu Chemical-Check Lab, WAK-NO2(C), WAK-NO3(C)), respectively. The absorbance was analyzed in each assay using a spectrophotometer (Kyoritsu Chemical-Check Lab, DPM-MTSP). The 1.5 mL of each solution needed to detect the chemical species was obtained by recollecting the mist in a 35 mm dish (AGC Techno Glass, 3000-035). We also dissolved the standard of the chemical species in each solution and performed the same colorimetric assay to confirm that there was no influence of coexisting chemicals produced by the plasma. In cases where the chemical species exceeded the upper limit of detection, the concentration of the species was determined by stepwise dilution by each solution. The effect of dilution on the chemical species concentration was checked beforehand using a standard solution. The pH and conductivity of each solution were measured using a pH meter (AS ONE, AS-pH-11B) and a conductivity meter (Horiba, LAQUAtwin-EC-33B).

## Results and discussion

### Plasma generation and its optical characteristics

**Figure 2:**
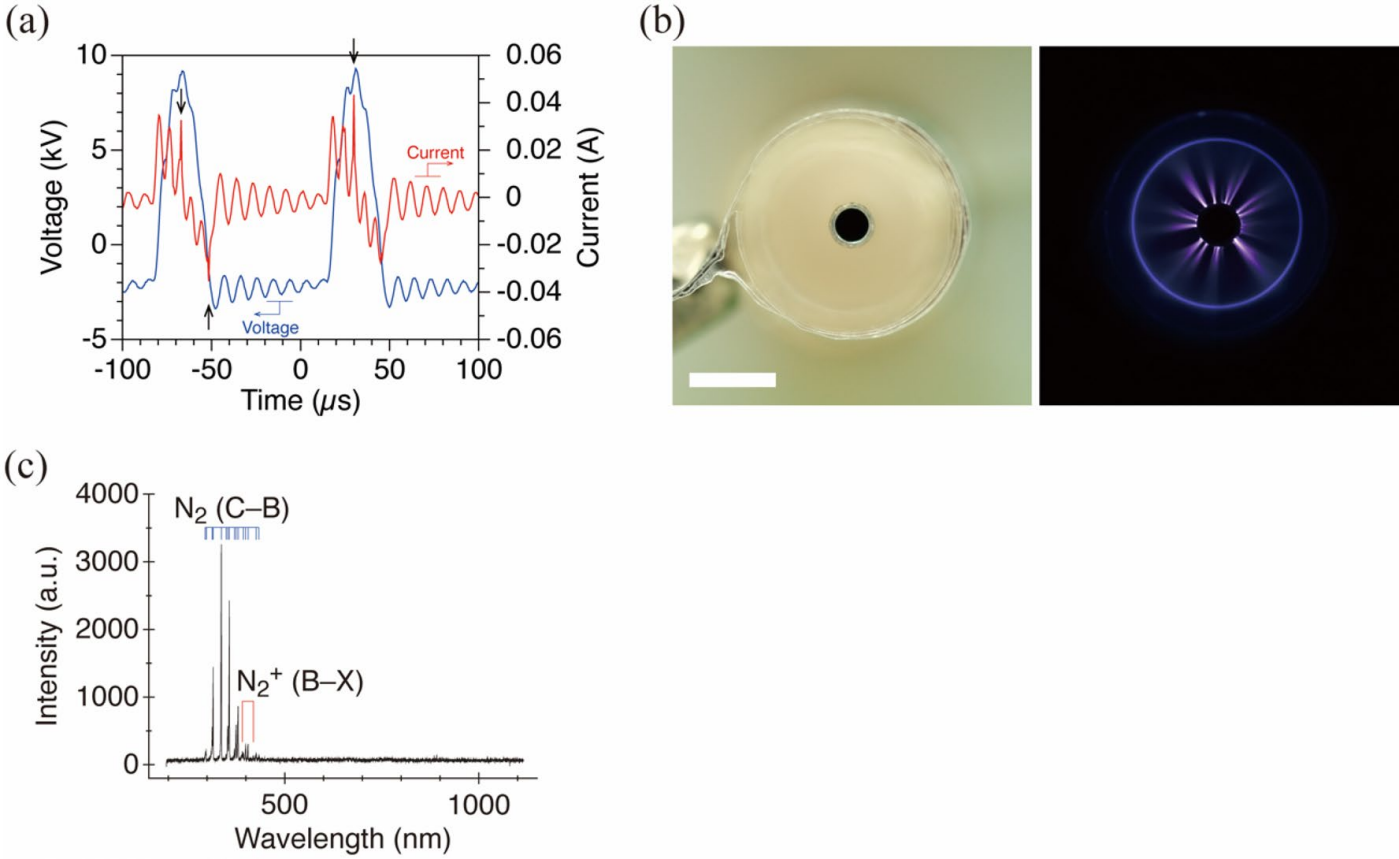
Characteristics of plasma generated by the DBD. (**a**) Waveforms of the applied voltage (blue) and discharge current (red). Black arrows indicate discharge current pulses. (**b**) Light emitted by plasma generated in the electrode unit when no solution was infused. Scale bar, 3 mm. (**c**) Emission spectrum of the plasma discharge. Typical peaks are attributed to the N_2_-SPS(C–B) and N_2_^+^-FNS(B–X).

The applied voltage started to increase from − 2.0 kV to + 9.3 kV in 15 µs (Fig. 2a). The voltage then decreased to − 3.2 kV, oscillated, and gradually rose to − 2 kV, and the next pulse was applied. The pulse width of the applied voltage was 30 µs. During the application of the voltage pulse, there were an oscillated displacement current of up to about 0.03 A and several current pulses corresponding to the plasma discharge. The discharge current pulses were seen in both positive and negative polarities, with a maximum of about 0.04 A (in absolute value).

Focusing on the plasma emission during discharge, we can see that streamers were generated from the needle, which was the driven electrode, towards the inner surface of the glass tube with the ground electrode (Fig. 2b). The points at which these streamers were formed varied intermittently (Supplementary Movie S1). The spectra of the plasma emission exhibited peaks (Fig. 2c), and they were attributed to the second positive system of nitrogen [N_2_-SPS; N_2_(C–B)] and the first negative system of nitrogen [N_2_^+^-FNS; N_2_^+^(B–X)]^19^. These emission peaks are commonly found in atmospheric pressure plasma discharge^20^.

The discharge appeared to be of a DBD type, based on the observation of the discharge current, the light emission, and optical characteristics.

### Dynamics and discharge characteristics in nano-sized mist

**Figure 3:**
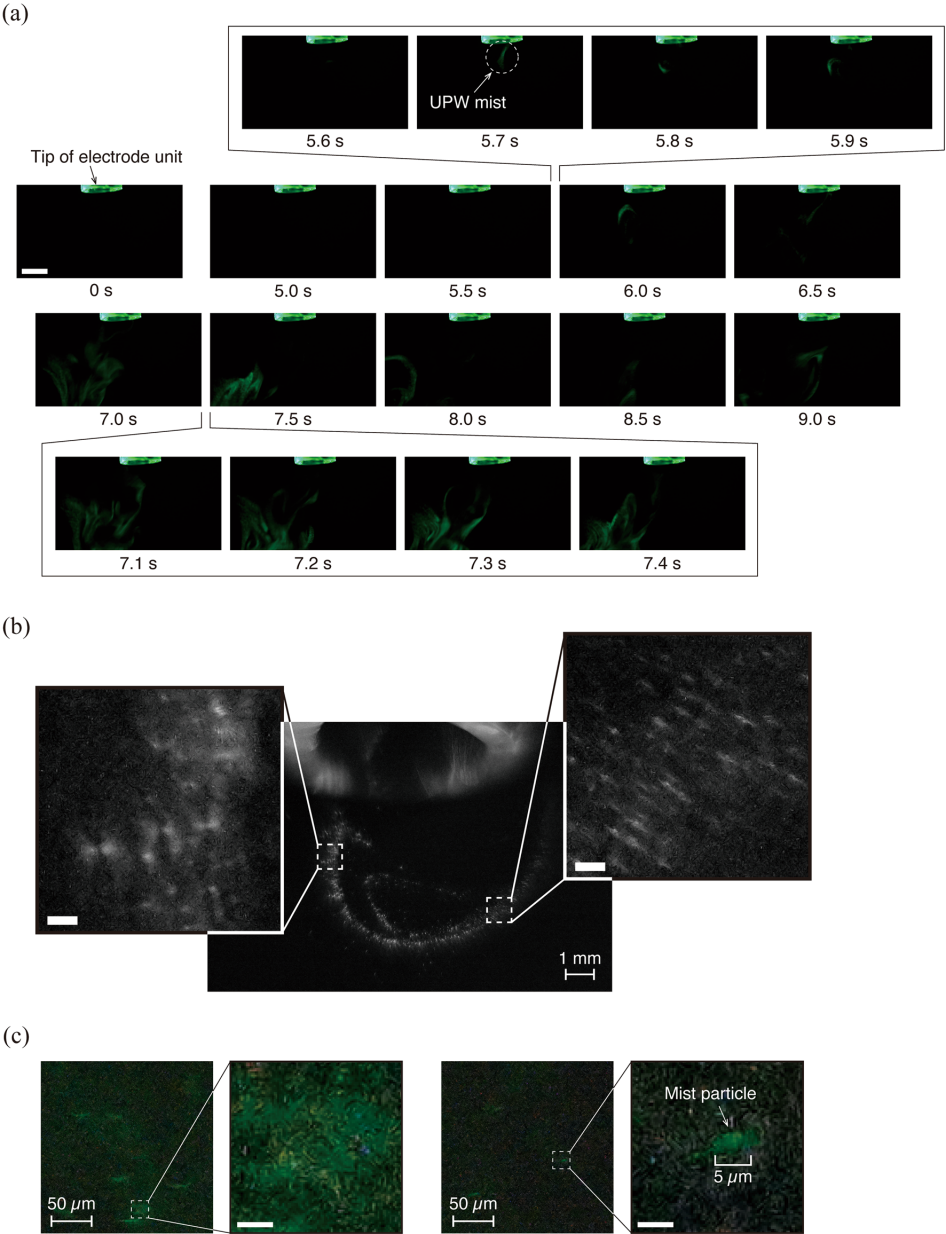
Nano-sized mist generated by passing a solution through DBD. (**a**) Time-course images of the UPW mist captured with a macro lens, visualized at 240 fps. The infusion rate of UPW was 50 µL/min. These images were obtained from Supplementary Movie 2. Scale bar, 5 mm. (**b**) Magnified high-speed images of the UPW mist taken with the 10 × microscope lens. Scale bars, 100 µm. (**c**) Magnified high-speed images of the UPW mist taken with the 100 × microscope lens. Scale bars, 5 µm. These images show a single frame taken at a shutter speed of 1/8000 s and an ISO sensitivity of 8000.

We then infused UPW into the dielectric barrier discharging electrode unit, and succeeded in generating a nano-sized mist to be blown out from the tip of the applying electrode (Fig. 3 and Supplementary Movie S2). Based on the images taken with a 10 × or 100 × microscope lens, we measured the pixel size for relatively large mist particles and calculated the actual particle size by scale calibration. Particles clearly visible ranged in size from 5 to 50 μm (Fig. 3b, c). This study has the disadvantage of capturing larger than the actual size because of the laser optical visualization technique. However, most of the mist particles was smaller than the minimum detectable limit [at least 400 nm or less; the resolution of the optical system with 100 × microscope lens used in this study (Fig. 3c)], indicating that many of them were potentially in the nanometer size range. The space where the plasma is generated, i.e., the field where the nano-sized mist is produced, has many molecules that are biased toward the same polar charge as the mist particles. After the droplets flowing out of the needle tip were atomized by Coulomb repulsion similar to electrostatic spray^21^, they may have interacted with the surrounding plasma (molecules biased toward the same polarity charge) and then changed into even finer particles. The efficiency of mist generation depended on the infusion rate of the solution. Although the mist was generated even under conditions of high infusion rate (≥ 500 µL/min for UPW and PBS; ≥ 100 µL/min for castor oil, respectively), droplet formation at the tip of the unit occurred preferentially (Supplementary Fig. S1). The discharge characteristics at the time of mist generation exhibit that a number of discharge current pulses were induced during applying the voltage pulse (Supplementary Fig. S2). The discharge current pulse was larger (up to about 0.06 A) than in the case where no solution was infused.

**Figure 4:**
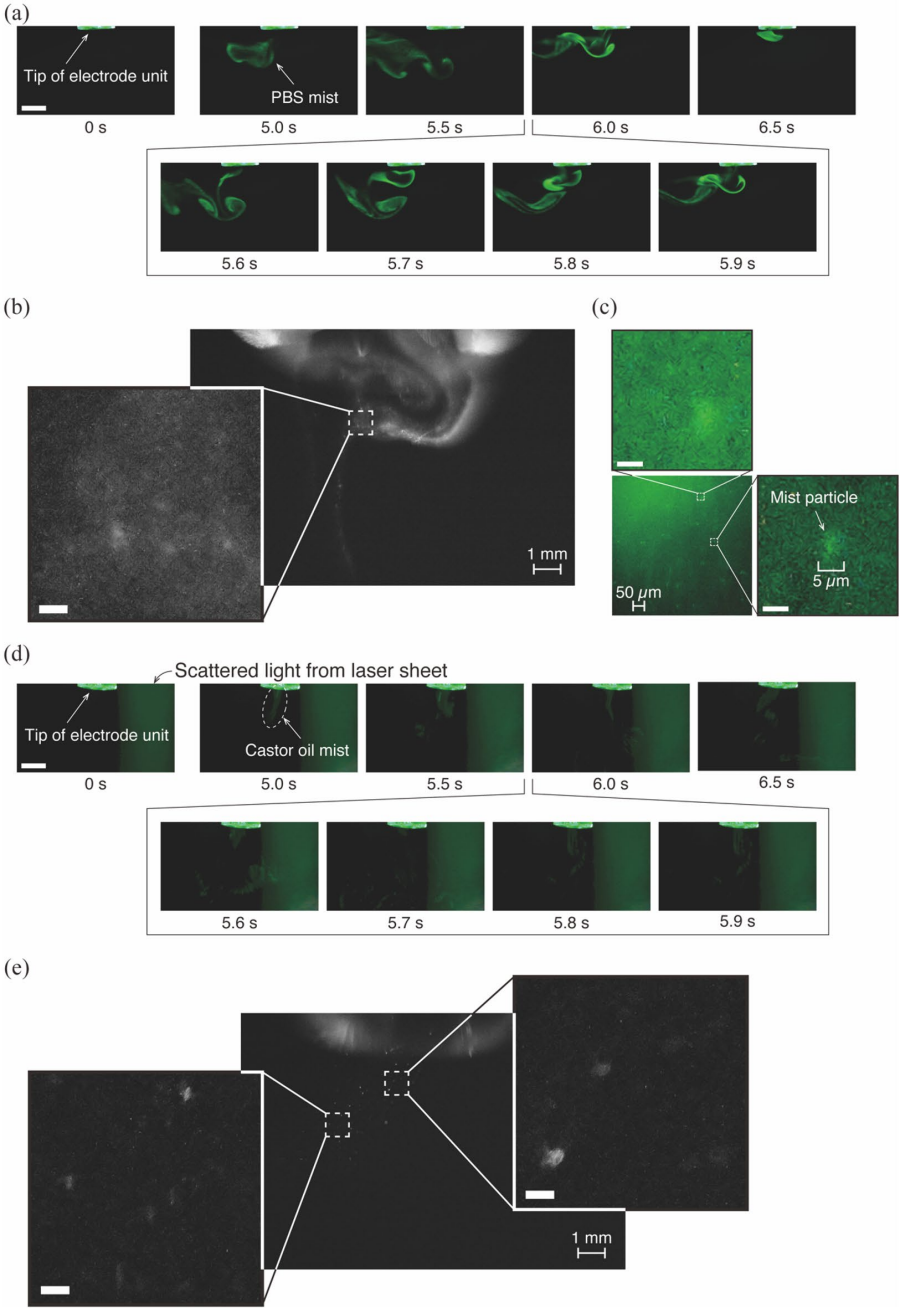
Nano-sized mist generated from each solution. Time-sequential images of the mist captured with a macro lens, visualized at 240 fps. The infusion rate of (**a**) PBS or (**d**) castor oil was 50 µL/min. These images were obtained from Supplementary Movies 3 and 4. Scale bar, 5 mm. Gamma correction is applied to the image (**d**) because the castor oil mist particles are too fine to be seen. Magnified images of the mist generated from (**b**) PBS or (**e**) castor oil, taken with the 10 × microscope lens. Scale bars, 100 µm. (**c**) Magnified high-speed images of the PBS mist taken with the 100 × microscope lens. Scale bars, 5 µm. These images show a single frame taken at a shutter speed of 1/8000 s and an ISO sensitivity of 8000.

**Figure 5:**
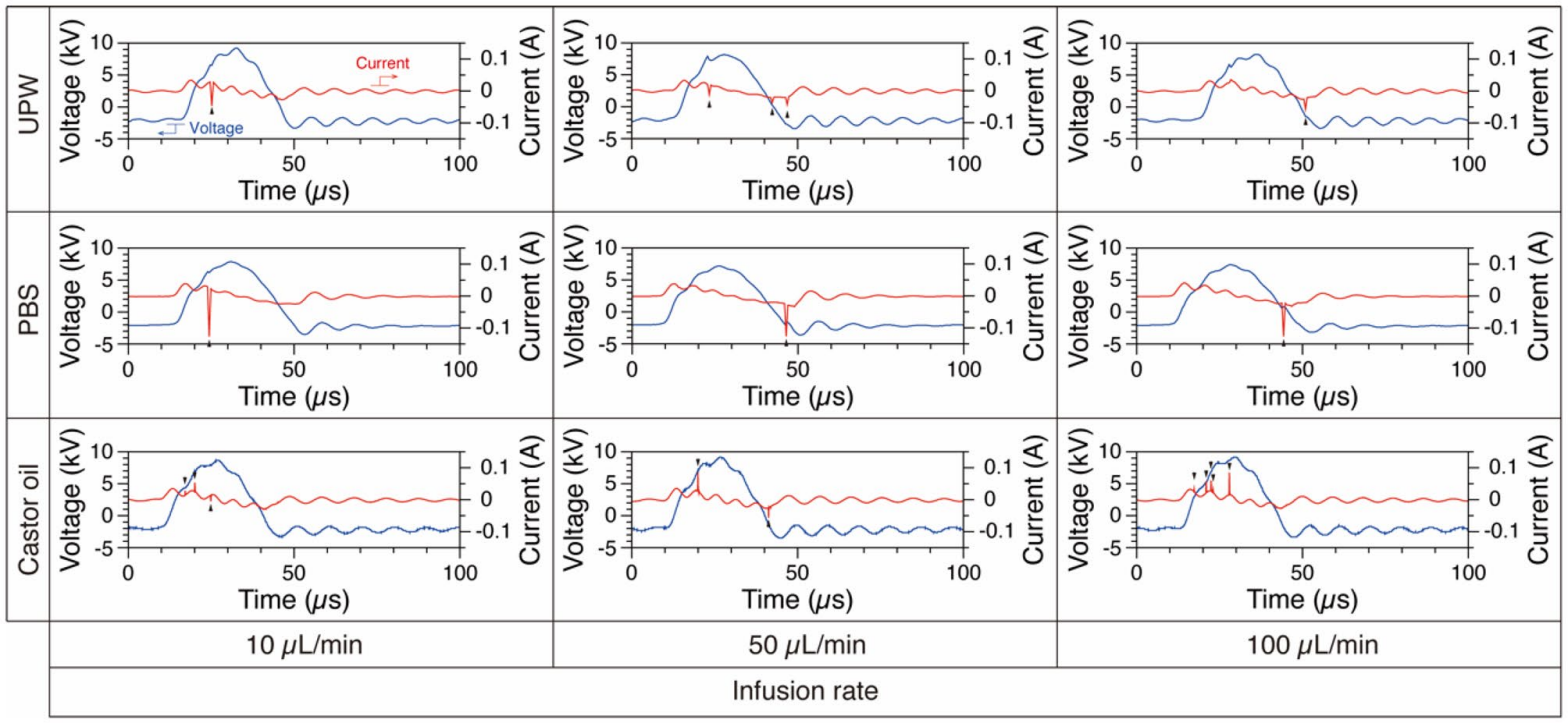
Comparison of waveforms of the applied voltage (blue) and discharge current (red) when each solution is infused at a flow rate capable of generating nano-sized mist. Black arrows indicate discharge current pulses. UPW, ultrapure water; PBS, phosphate buffered saline.

The developed nano-size mist generator can be applied to atomization not only of UPW but also of PBS with high electrical conductivity and castor oil with high viscosity (Fig. 4 and Supplementary Movies S2, S3, and S4). There was no significant difference in the conditions suitable for mist generation with respect to the type of solution. We could see some differences in the generation efficiency of mist itself and its finer size depending on the solution type, but it was possible to produce a mist potentially in the nanometer size range. The discharge characteristics of each solution in mist generation showed that discharge current pulses occurred as in the case of UPW (Fig. 5). The pulses in PBS were larger than those in the other solutions, exceeding 0.1 A. In PBS, the pulse width of the applied voltage was stretched by about 5 µs, and the oscillations in both voltage and current were smaller. The pulse widths of the discharge current for castor oil were, in contrast, smaller than those of the other solutions. These differences in the discharge characteristics might be due to the electrical conductivity^22^. This notion is supported by the fact described below that there is a marked difference in physical and chemical characteristics between UPW or castor oil, which has a low conductivity^23,24^, and PBS with a high conductivity. Moreover, the changes in electrical conductivity of UPW by generation of chemical species induced a slight difference in the discharge characteristics between UPW and castor oil. We attribute that the physicochemical characteristics and the plasma-generated chemical species caused differences in the discharge characteristics and ease of generating fine mist of each solution.

### Physical and chemical characteristics of solutions from which the nano-sized mist was produced

**Figure 6:**
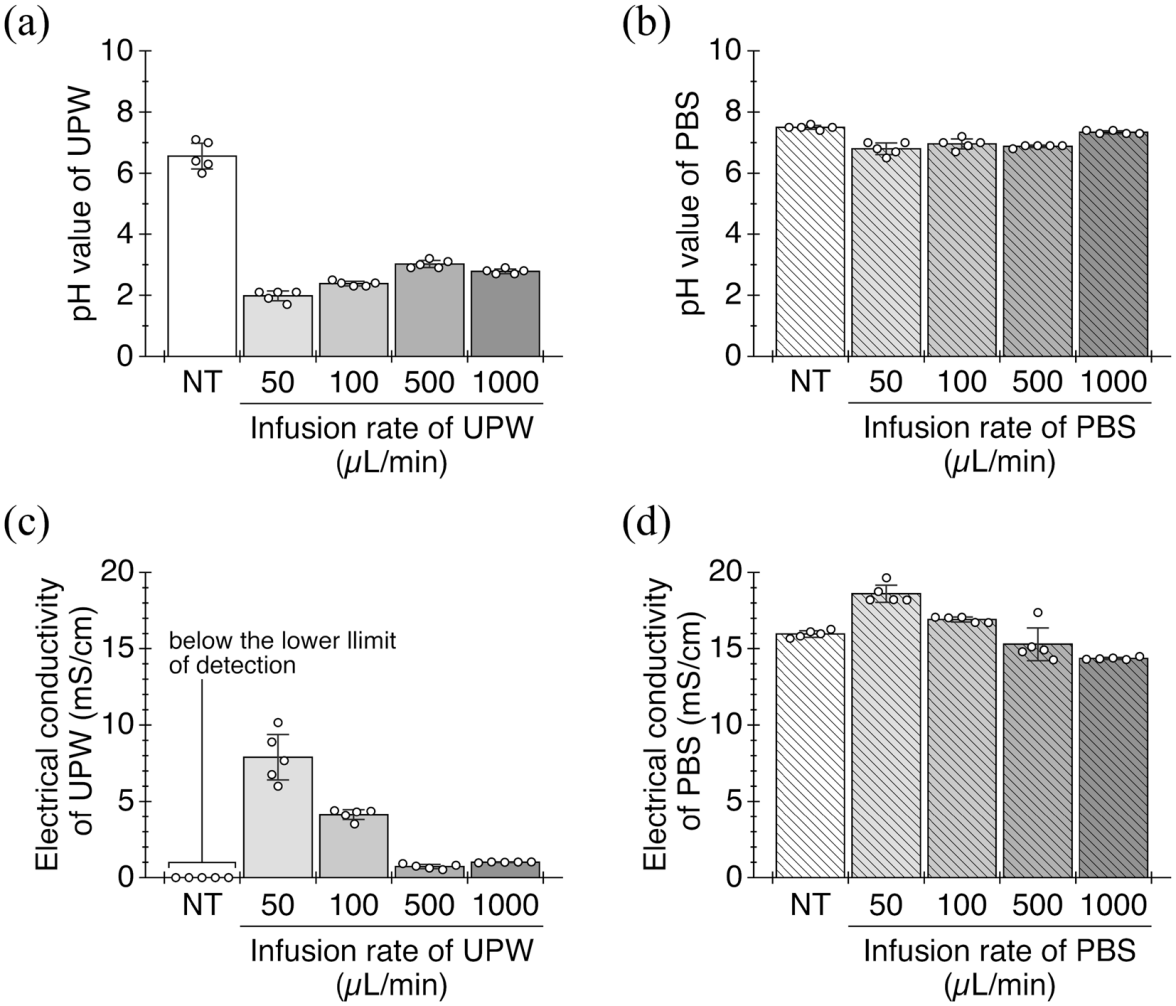
Physical characteristics of the nano-sized mist generated by the DBD. (**a, b**) The pH values of UPW and PBS before and after passing through the plasma discharge at each infusion rate (mean ± SD, *n* = 5). (**c, d**) Electrical conductivity of UPW and PBS before and after passing through the plasma discharge at each infusion rate (mean ± SD, *n* = 5). UPW, ultrapure water; PBS, phosphate buffered saline. NT, samples without plasma treatment (no treatment).

After passing UPW through the DBD, its pH value decreased from 6.5 to below 3.0, with some differences depending on the infusion rate (Fig. 6a). In contrast, the pH of PBS slightly decreased from 7.4 to about 6.8 due to the interaction with the plasma (Fig. 6b). The conductivity of each solution tended to increase with changes in its pH value, and reached the maximum value of 7.9 mS/cm (UPW, Fig. 6c) or 18.6 mS/cm (PBS, Fig. 6d), respectively, at the infusion rate of 50 µL/min. The changes in these characteristics can be attributed to the influence of chemical species produced in each solution by interaction with the plasma.

**Figure 7:**
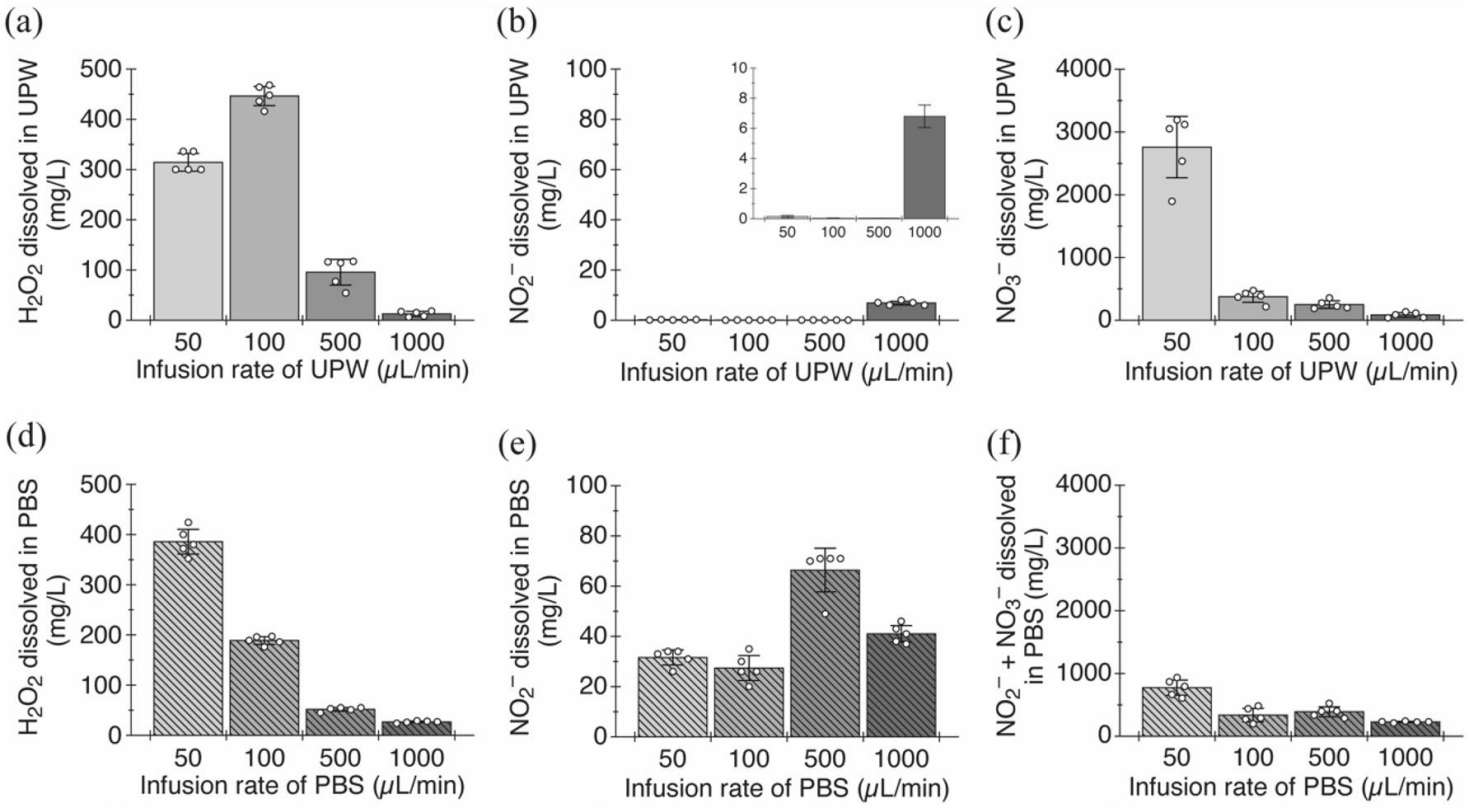
Chemical species in the nano-sized mist generated by the DBD. Concentrations of dissolved (**a, d**) H_2_O_2_, (**b, e**) NO_2_^−^, (**c**) NO_3_^−^, and (**f**) NO_2_^−^ + NO_3_^−^ in UPW or PBS after passing through the plasma discharge at each infusion rate (mean ± SD, *n* = 5). UPW, ultrapure water; PBS, phosphate buffered saline.

Various chemical reactions occurred at the gas–liquid interface where plasma discharge occurs, leading to the formation of oxidative products in the solution^18^. Our plasma source also produced oxidative compounds such as hydrogen peroxide, nitrite ion, and nitrate ion in each solution (Fig. 7). The amount of hydrogen peroxide produced in UPW increases with decreasing its infusion rate, reaching a maximum value (446.4 ± 19.0 mg/L) in the rate of 100 µL/min, and the produced amount (314.4 ± 17.6 mg/L) decreases under the condition with the lowest rate (50 µL/min) (Fig. 7a). Hydrogen peroxide is produced by the coupling of hydroxyl radicals, which are generated from water molecules in solution by the plasma, as shown in the following chemical reaction^25^.

$${\text{OH }} \cdot \, + {\text{ OH }} \cdot \, \to {\text{ H}}_{{2}} {\text{O}}_{{2}}$$

Hydrogen peroxide also acts as an oxidant under acidic conditions as followed^26,27^.

$${\text{H}}_{{2}} {\text{O}}_{{2}} + {\text{ 2H}}^{ + } + {\text{ 2e}}^{ - } \to {\text{ 2H}}_{{2}} {\text{O}}$$

This half-reaction of hydrogen peroxide as an oxidant might induce a decrease in its produced amount at the 50 µL/min condition. This notion is supported by the result that the concentration of hydrogen peroxide in PBS, which had a small change in pH value, monotonically increased with decreasing the infusion rate (Fig. 7b). On the other hand, nitrite and nitrate ions are produced due to the reaction of nitric oxide, nitrogen dioxide, and hydroxyl radicals generated by the plasma, as shown in the following reactions^28^.

$$\begin{aligned} & {\text{NO }} + {\text{ OH }} \cdot \, + M \to {\text{ HNO}}_{{2}} + M \\ & {\text{NO}}_{{2}} + {\text{ OH }} \cdot \, + M \to {\text{ HNO}}_{{3}} + M \\ & {\text{2NO}}_{{2}} + {\text{ H}}_{{2}} {\text{O }} \to {\text{ HNO}}_{{3}} + {\text{ HNO}}_{{2}} \\ \end{aligned}$$

Here, *M* indicates the third body, which is typically H_2_O. The generated nitric acid and nitrous acid dissociate in the solution to yield nitrite and nitrate ions^29^.

$$\begin{gathered} {\text{HNO}}_{{2}}\,\leftrightarrows\,{\text{H}}^{ + } + {\text{ NO}}_{{2}}^{ - } \hfill \\ {\text{HNO}}_{{3}} \, \leftrightarrows \,{\text{H}}^{ + } + {\text{ NO}}_{{3}}^{ - } \hfill \\ \end{gathered}$$

The smaller the infusion rate of the solution, the more nitrogen compounds were produced, with some exceptions. At the rate of 1000 µL/min, the concentration of the produced nitrite ions was 6.80 ± 0.75 mg/L in UPW (Fig. 7c) or 41.0 ± 3.3 mg/L in PBS (Fig. 7d), while it was less than 0.15 mg/L under other rate conditions. Nitrate ions were most abundantly produced under the condition of 50 µL/min solution infusion rate, and their concentrations were 2759.2 ± 489.5 mg/L in UPW (Fig. 7e). In PBS, the amount of nitrite ions produced was large, and they must be reduced and converted to nitrate ions to measure the produced amount of nitrate ions independently. The reduction agent was affected by the hydrogen peroxide produced at the same time, making it difficult to remove nitrite ions. We therefore measured the total amount of nitrite and nitrate ions generated in PBS. As a result, their concentration was 774.0 ± 122.1 mg/L (Fig. 7f). The reason why there are fewer oxidative compounds produced by the plasma in PBS than those in UPW is probably because phosphoric acid compounds such as the disodium hydrogen phosphate and potassium dihydrogen phosphate in PBS acted as scavengers for them. The difference in the amount of nitrite and nitrate ions produced in UPW and PBS might depend on the presence or absence of buffering related to changes in pH value of each solution.

## Conclusion

We demonstrated the method to directly generate nano-sized mist by passing a solution through DBD. We succeeded in producing mists potentially in the nanometer size range for three types of solutions: UPW, PBS (with high electrical conductivity), and castor oil (with electric non-conductance and high viscosity). The possible mechanism for the formation of nano-sized mist is Coulomb repulsion and the interaction with charge polarity caused by the plasma. The ideal infusion rate of the solutions was less than 100 µL/min under the voltage condition applied in this study (12.5 kV from peak to peak, with a frequency of 10 kHz). In these conditions, almost all liquid flowed out of the developed generator as mist. Ease of fine mist generation of these solutions was influenced by the plasma-induced changes in their physical and chemical characteristics. We expect that our technology, which can turn water-soluble or fat-soluble compounds dissolved in a solvent into a nano-sized mist, will be applied to the transdermal drug delivery system. Applied voltage and electric power might affect the efficiency of mist generation. Since the applied voltage was fixed in this study, there is room for further study about their effects on mist generation and transdermal absorption.

The developed nano-sized mist generator can cause atomization of a solution without grounding the target being sprayed. The point about grounding is the biggest difference from electrostatic spray used for mist generation. Electrostatic spray has a risk of large currents flowing into the target, whereas this method does not have such a risk. However, the spraying capability of this method on the target is weak, so we need to introduce a mechanism to guide the mist by airflow. On the other hand, not grounding the target might keep the charged state of the sprayed mist particles, albeit temporarily. It is unclear how the charged mist influences the enhancement of transdermal absorption, but we hope to clarify its details in future work.

## Supplementary Information

Supplementary Video 1.

Supplementary Video 2.

Supplementary Video 3.

Supplementary Video 4.

Supplementary Information.

## Data Availability

The authors declare that all data supporting the findings of this study are available within this article and its supplementary information files or from the corresponding author upon reasonable request.
