# Supplementary material for: Potential generation of nano-sized mist by passing a solution through dielectric barrier discharge
Source: Sci Rep. 2022 Jun 22;12:10526. doi: 10.1038/s41598-022-14670-4 (PMC9217796; doi:10.1038/s41598-022-14670-4)
Supplement: Supplementary file 5 — Supplementary Information. [file 41598_2022_14670_MOESM5_ESM.pdf]

Supplementary Information

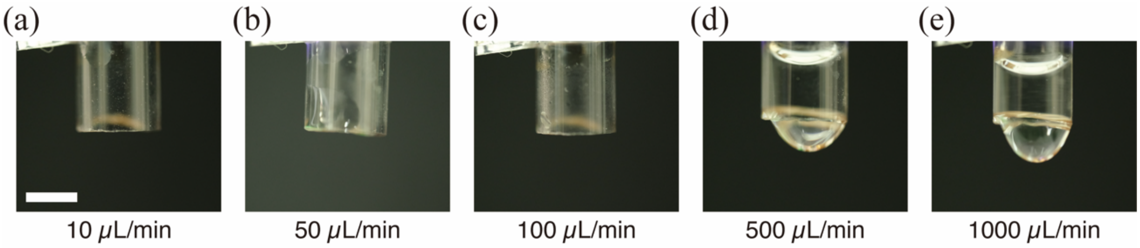

Supplementary Figure S1. Tip of the electrode unit during nano-sized mist generation at each infusion rate of UPW. (a) 10 μL/min, (b) 50 μL/min, (c) 100 μL/min, (d) 500 μL/min, and (e) 1,000 μL/min. At low infusion rates (a–c), the generated nano-sized mist adhered to the inner surface of the glass tube, and the tube fogged up. High infusion rate condition (d, e), in contrast, induced to form droplets at the tip of the electrode unit rather than mist formation. Scale bar, 5 mm.

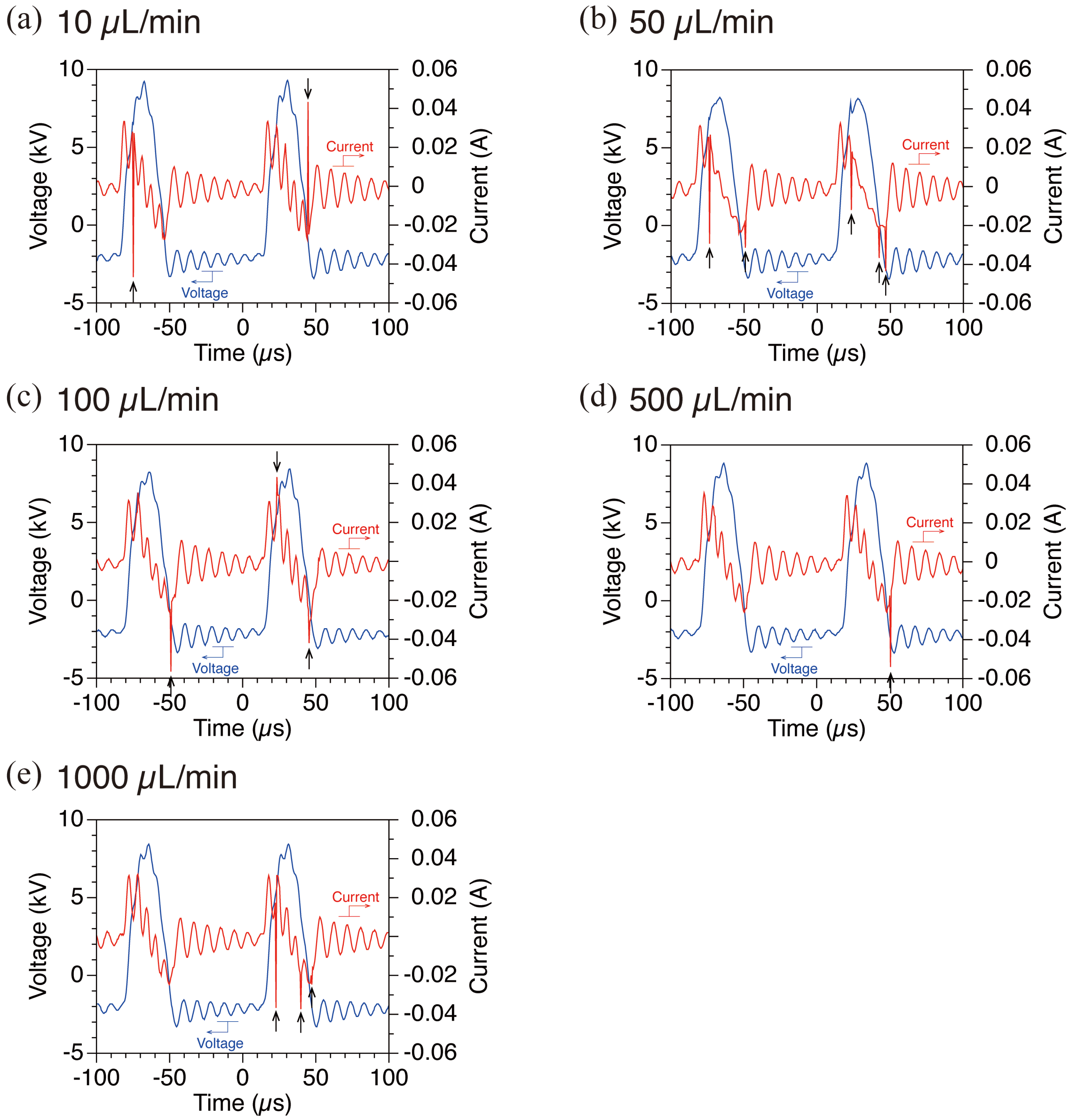

Supplementary Figure S2. Representative waveforms of the applied voltage (blue) and discharge current (red) when UPW was infused at each flow rate. (a) 10 μL/min, (b) 50 μL/min, (c) 100 μL/min, (d) 500 μL/min, and (e) 1000 μL/min. Black arrows indicate discharge current pulses.

Description of Additional Supplementary Files

File Name: Supplementary Movie S1
Description: Streamer generation during plasma discharge in the electrode unit. Movie was captured at the frame rate of 60 fps, ISO sensitivity of 2500, and the diaphragm of the camera of 2.8. Scale bar = 5 mm.

File Name: Supplementary Movie S2
Description: Spraying dynamics in nano-sized mist of UPW. The infusion rate of UPW was 50 μL/min. Mist begins to blow from the tip of the electrode about 6 s into the movie. The movie was captured at the frame rate of 240 fps, ISO sensitivity of 2500, and the diaphragm of the camera of 2.8. Scale bar = 5 mm.

File Name: Supplementary Movie S3
Description: Spraying dynamics in nano-sized mist of PBS. The infusion rate of PBS was 50 μL/min. Mist begins to blow from the tip of the electrode about 1 s into the movie. The movie was captured at the frame rate of 240 fps, ISO sensitivity of 2500, and the diaphragm of the camera of 2.8. Scale bar = 5 mm.

File Name: Supplementary Movie S4
Description: Spraying dynamics in nano-sized mist of castor oil. The infusion rate of castor oil was 50 μL/min. Mist begins to blow from the tip of the electrode about 2 s into the movie. The movie was captured at the frame rate of 240 fps, ISO sensitivity of 2500, and the diaphragm of the camera of 2.8. Scale bar = 5 mm.
